# G-CSF and Exenatide Might Be Associated with Increased Long-Term Survival of Allogeneic Pancreatic Islet Grafts

**DOI:** 10.1371/journal.pone.0157245

**Published:** 2016-06-10

**Authors:** Alessia Zoso, Paolo Serafini, Giacomo Lanzoni, Eduardo Peixoto, Shari Messinger, Alejandro Mantero, Nathalia D. Padilla-Téllez, David A. Baidal, Rodolfo Alejandro, Camillo Ricordi, Luca Inverardi

**Affiliations:** 1 Diabetes Research Institute, University of Miami, Miami, Florida, United States of America; 2 Microbiology Immunology Department, University of Miami, Miami, Florida, United States of America; 3 Department of Public Health Sciences, University of Miami, Miami, Florida, United States of America; Children's Hospital Boston/Harvard Medical School, UNITED STATES

## Abstract

**Background:**

Allogeneic human islet transplantation is an effective therapy for the treatment of patients with Type 1 Diabetes (T1D). The low number of islet transplants performed worldwide and the different transplantation protocols used limit the identification of the most effective therapeutic options to improve the efficacy of this approach.

**Methods:**

We present a retrospective analysis on the data collected from 44 patients with T1D who underwent islet transplantation at our institute between 2000 and 2007. Several variables were included: recipient demographics and immunological characteristics, donor and transplant characteristics, induction protocols, and additional medical treatment received. Immunosuppression was induced with anti-CD25 (Daclizumab), alone or in association with anti-tumor necrosis factor alpha (TNF-α) treatments (Etanercept or Infliximab), or with anti-CD52 (Alemtuzumab) in association with anti-TNF-α treatments (Etanercept or Infliximab). Subsets of patients were treated with Filgrastim for moderate/severe neutropenia and/or Exenatide for post prandial hyperglycemia.

**Results:**

The analysis performed indicates a negative association between graft survival (c-peptide level ≥ 0.3 ng/ml) and islet infusion volume, with the caveat that, the progressive reduction of infusion volumes over the years has been paralleled by improved immunosuppressive protocols. A positive association is instead suggested between graft survival and administration of Exenatide and Filgrastim, alone or in combination.

**Conclusion:**

This retrospective analysis may be of assistance to further improve long-term outcomes of protocols for transplant of islets and other organs.

## Introduction

Type 1 Diabetes (T1D) treatments aim to improve patients’ quality of life by controlling glycemic levels, avoiding hypoglycemia, and preventing diabetes-associated complications. Despite substantial improvements in pharmacological diabetes therapy and technical advances in blood glucose monitoring and insulin application, a subset of patients fail to meet the target parameters. For patients with T1D and problematic hypoglycemia, islet transplantation is a promising therapy and has several advantages including: (i) provides physiological glucose regulation, resolution of impaired hypoglycemia awareness, prevents severe hypoglycemia and in many instances insulin independence; (ii) it reduces morbidity and mortality associated with pancreas transplantation [1,2]. The effectiveness and reproducibility of islet transplantation has been proven worldwide [3]. However, inconsistent results among centers have been observed due to many confounding factors, including: quantity/quality of islets, autoimmune reactivation, toxicity of immunosuppressive regimens, and transplantation site [1]. In an attempt to improve transplant outcomes, retrospective analysis of registry data as well as single-center studies represent important contributions to identify the most effective therapeutic options and improve effectiveness. Here, we report a retrospective analysis of 15 years’ experience in implementing islet transplantation as a treatment option in patients with high glycemic lability, severe hypoglycemia, and hypoglycemia unawareness.

## Research Design and Methods

### Subjects and protocol

The analysis included data from patients with T1D (age 18–65; n = 44; duration>5 years), with negative basal or stimulated C-peptide (<0.3ng/ml), hypoglycemia unawareness, severe hypoglycemia, and labile diabetes who underwent pancreatic islet transplantation between 2000 and 2007. Institutional Review Board (IRB) approval and written informed consent were obtained prior to study initiation (ClinicalTrials.gov: NCT00021801/IRB#20000024, NCT00306098/IRB#20000196, NCT00315588/IRB#20000329, NCT00014911/IRB#20000658, NCT00315627/IRB#20000205, NCT00315614).

Immunosuppressive regiments and transplant protocols have been described previously [4–9]. Briefly, Daclizumab (Zenapax^®^, Roche): five-doses of 1mg/kg biweekly on transplant day follow by administration of Tacrolimus (Prograf^®^, Fujisawa; trough levels 4–6 ng/ml), and Sirolimus (Rapamune^®^, Wyeth Pharmaceuticals-Inc.; trough levels 12–15 ng/mL for 3 months; 10–12 ng/ml thereafter). Alemtuzumab (Lemtrada^®^, Genzyme): intravenous (i.v.) injections of 20mg post-infusion (day 1 and 0). Premedication with diphenhydramine (50mg, i.v.), acetaminophen (650 mg, orally), and methylprednisolone (125mg, i.v) were required. Steroids were not administered with the second dose of Alemtuzumab unless clinically indicated. All patients underwent maintenance of immunosuppression with Sirolimus (trough levels 7 to 10 ng/ml) and Tacrolimus (trough levels 4 to 6 ng/ml). Six patients had to discountinued Sirolimus treatment due to severe side effects (ovarian cysts n = 1; mouth ulcers n = 1; peripheral edema, n = 4), and continued immunosuppression maintenance with Tracolimus and mycophenolic acid (Myfortic^®^, Novartis) up to a total dose of 720 mg two times per day as clinically tolerated. In one subject Sirolimus was discontinued by nephrologist’s choice since subject was already on Tacrolimus and MMF. Infliximab (Remicade^®^, Centocor): 5 mg/kg i.v. 2h prior to the first infusion. Etanercept (Enbrel^®^, Immunex Corporation): 50 mg i.v. within 1h of islet infusion and 25 mg subcutaneously twice a week for the following 2 weeks.

Exenatide (Byetta^®^, Amylin) 5 μg twice a day, concomitant with the two largest meals of the day. Dose was increased or decreased as tolerated up to three injections and a target total daily dose (TTD) of 30 μg. At time of commencement of Exenatide, insulin dose was reduced by 30% to 40%. Filgrastim (Neupogen^®^, Amgen): 300μg subcutaneously (one or several doses per event).

Insulin Independence was defined as hemoglobin-A1c (HbA1c) ≤ 6.5%, without exogenous insulin administration, and capillary fasting and postprandial blood glucose levels were ≤ 7.8 mmol/l (140mg/dl) and ≤ 10 mmol/l (180 mg/dl), respectively.

Graft dysfunction was defines as: C-peptide-positivity, fasting capillary glucose >7.8 mmol/l and/or postprandial capillary glucose >10.0 mmol/l in 3 occasions in any 7-day period; reintroduction of exogenous insulin.

Graft failure was defined as negative stimulated C-peptide (≤ 0.3 ng/ml) during post-transplant Mixed-meal tolerance test (MMTT) follow-up.

Pancreata were obtained from cadaveric donors, 15–65 years of age, with <10 minutes warm ischemia time. Islets were isolated using a modified automated method and purified using continuous gradients (Ficoll/Biocoll). Isolated islets were maintained in Miami defined media (CellGrowth) at 5% CO2 at 37°C for 24h and 22°C up to 72h from the isolation. After standard product release testing, islets were transplanted using the bag infusion technique via the percutaneous transhepatic intraportal route.

### Statistical Analysis

Continuous and discrete variables were collected, and analyzed with bivariate analysis to determine their associations with graft survival. Discrete variables were compared using log rank tests. For continuous variables, Cox models were fit with corresponding tests of significance for the effect of the predictor on the hazard for graft failure. Potentially confounding factors were identified as those having bivariate associations with both graft survival as well as drug-treated group. Where possible, adjustment for potential confounders was investigated but this was constrained due to sample size.

## Results

### Subjects

Forty-four long-standing patients with T1D (mean 29.5 years, Standard Error of the Mean (SEM) 1.7 years) underwent one or multiple islet transplantation(s) (Table 1).

**Table 1:** Baseline patient characteristics, induction protocols, and additional medical treatments.

**Demographic data**	**Mean ± SD**	**Range value**
Age (years)	55±7.8	38–74
CMV positivity	45%	
Body weight (kg)	64.7 ± 11.4	47.5–98
BMI (kg/m^2^)	24.5 ± 3.4	21.6–23.6
Sex (n = M/F)	18/26	
Duration of type 1 diabetes till 1^st^ infusion (years)	29.4 ± 11.4	7–48
Age at diagnosis (years)	13.56 ± 9.7	2–50
Fasting c-peptide before infusion	0.12 ± 0.037	0.1–0.3
Total Number of Infusion received	1.79±0.79	1–4
IEQ/kg (total amount received)	13.2x10^3^±6.3x10^3^	4.7x10^3^-34.1x10^3^
**Induction Treatments**	**Number of patients**	**%**
Daclizumab	13	29.5%
Daclizumab + anti-TNF-α treatments	25	56.8%
Daclizumab + Etanercept	7	15.9%
Daclizumab + Infliximab	17	38.6%
Daclizumab + Infliximab + Etanercept	1	2.3%
Alentuzumab + anti-TNF-α treatments	6	13.6%
Alentuzumab + Etanercept	5	11.4%
Alentuzumab + Infliximab + Etanercept	1	2.3%
**Additional medical treatments**		
None	13	30%
Exenatide	15	34%
Filgrastim	7	16%
Exenatide + Filgrastim	9	20%

**Fig 1:**
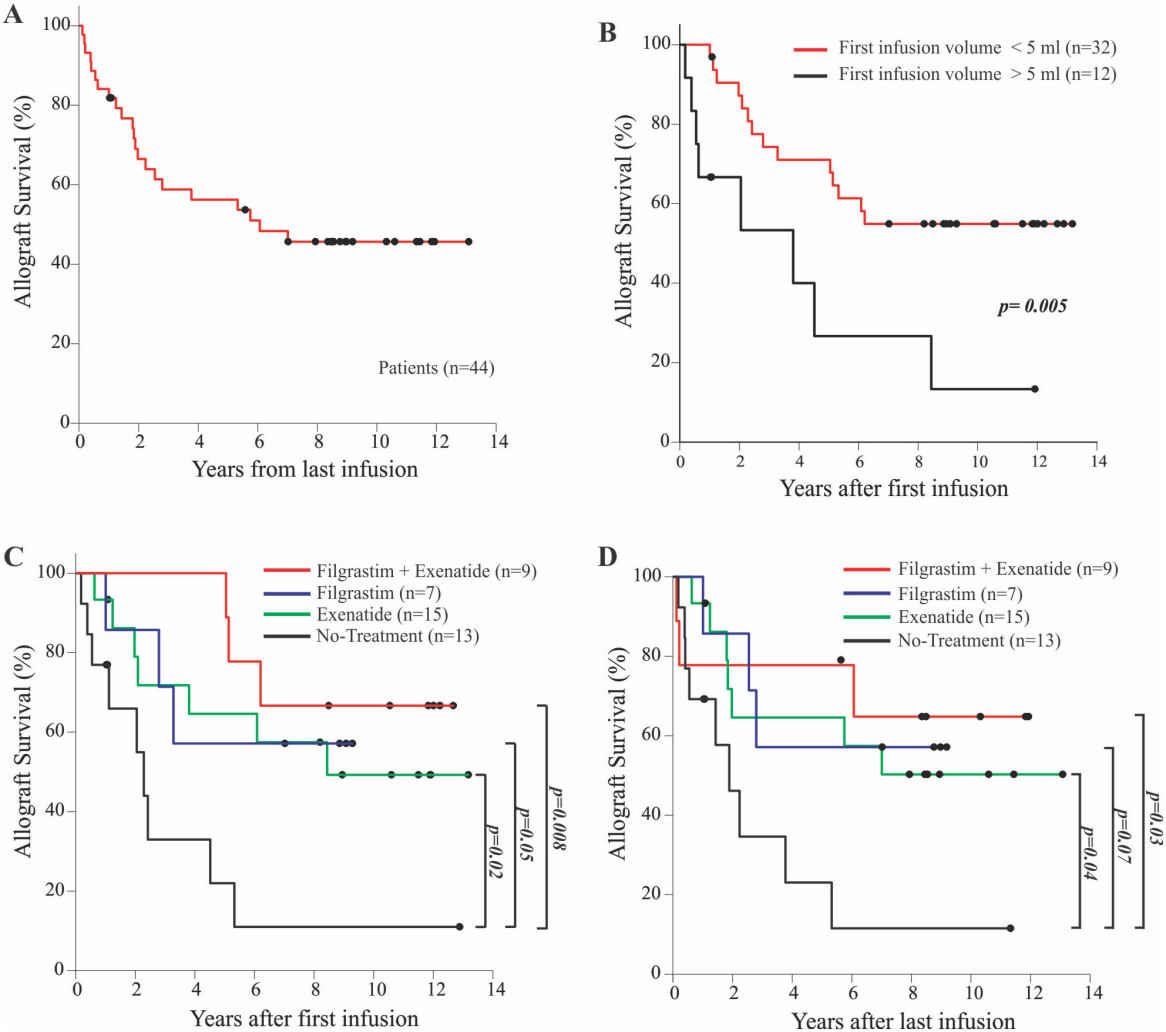
Allogeneic pancreatic islets transplant survival rate at the DRI. **A)** Overall allograph survival of the 44 T1D subjects underwent allogeneic islet transplantation. **B)** Allograft survival based on infusion volume used. Allograft survival after first (**C**) and last infusion (**D**) based on drug treatments received. Log-rank analysis was used.

Patients were insulin dependent, had undetectable stimulated C-peptide, history of highly problematic diabetes control, hypoglycemia unawareness, and severe metabolic lability. Overall, 66.3% of patients gained insulin independence. Mean graft survival from last infusion was 6.6 years (SEM = 0.8 years) with approximately 45% of patients with C-peptide levels above 0.3 ng/ml for more than 10 years (Fig 1A and S1 Fig). Islets (mean 860.9x10^3^, SEM 64.7x10^3^ Islet Equivalents, IEQ) from one or multiple donors were transplanted intraportally.

Progressive improvements in human islet isolation and purification techniques [10–12] have paralleled a reduction over the years of the final volume of islet cells infused, from more than 5ml (mean 7.75ml, SEM 0.77ml, n = 9) in the first years, to less than 5ml (mean 2.59ml, SEM 0.168ml, n = 35; Fig 1B and S2 Fig) in later years. Induction protocols to prevent islet rejection were also modified (Table 1) over the years: 1) Daclizumab alone, 2) Daclizumab and anti-TNF-α (Etanercept and/or Infliximab), or 3) Alemtuzumab and anti-TNF-α (Etanercept or Infliximab). As consequence of the immunosuppressive treatment, 36.3% (16/44) of the patients experienced moderate to severe neutropenia that was treated with Filgrastim. Nine of these 16 patients also received Exenatide to improve post-prandial glycaemia. 15 subjects received only Exenatide and 13 patients did not received either treatment (Table 1). Exenatide was given at the time of the initial (n = 4) or supplemental islet infusion (n = 4) [4].

### Bivariate analysis: effect of different treatments on graft survival

To evaluate whether demographic variables, clinical treatments, immunological aspects, or transplant characteristics influence graft survival, bivariate analyses were performed. At the end of the follow-up period, prolonged graft survival was associated with Exenatide/Filgrastim treatment when compared with no treatment: 45% survival in Exenatide only (hazard ratio 0.344; 95% confidence interval, 0.12475 to 0.9500, p = 0.0395), 57% in Filgrastim only (hazard ratio 0.2902; 95% confidence interval, 0.07686 to 1.0956, p = .0680), and 66% in Exenatide and Filgrastim (hazard ratio 0.2321; 95% confidence interval, 0.06087 to 0.8849; p = 0.0324) treated patients, suggesting a combined effect. Only 7% survival was observed in patients who did not receive either drug (Fig 1C and 1D).

No statistically significant associations (p≥0.05) were observed for other demographic and clinical parameters considered. In particular no significant association were found with patient’s age, body mass index, and duration of diabetes. Similarly, no association were found between graft survival and the number (p = 0.48) or the purity (p = 0.10) of transplanted islets, or the number of islets infusion that the patient received (p = 0.55).

Immunosuppressive induction protocols have been modified over the years to reduce adverse events and improve outcomes [13]. Interestingly, the addition of anti-TNF-α (Etanercept and/or Infliximab) to Daclizumab does not significantly affect graft survival (p = 0.44; S1A and S1B Fig). Anti-TNF-α in combination with Alemtuzumab may favor graft survival, and appears to be superior to the one with anti-CD25/anti-TNF-α antibodies. However, due to the fact that the study was underpowered, it failed to reach statistical significance (S1C Fig). These results are comparable to those obtained using T-cell depleting agents and anti-TNF-α antibodies [14]. Moreover, considering the depleting role of Daclizumab on T regulatory cells (Treg) [15], its possible substitution with anti-CD52 warrants further investigation.

We also observed that the first islet infusion volume used may influence graft survival: higher infusion volumes (≥ 5 ml), are associated with increased risk of graft failure (hazard ratio, 2.802; 95% confidence interval, 1.151 to 6.818; p = 0.0232; Fig 1B). Similar results were obtained when the average injected volume was evaluated (data not shown). These data confirm previous studies showing that high infusion volume is associated with surgical complications and primary non-function [16,17]while low volumes ameliorate islet bio-distribution, viability and functionality [18].

### Limitations of the bivariate analysis and confounding variables

**Fig 2:**
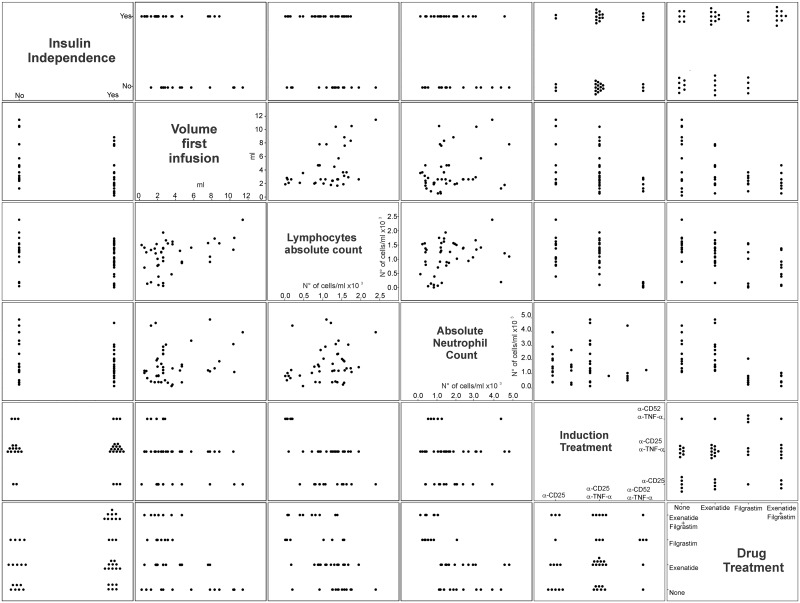
Analysis of the collinearity between selected variables. Scatter plot matrix representation has been used to identify possible correlation between the different variables that showed statistically significant (p≤0.05) association with allograph survival.

Because of the retrospective nature of this study, there are many inherent confounding factors that complicate estimation of independent associations with graft survival. Pair comparison analysis was performed to highlight possible correlations between drug treatment, insulin independence, induction treatments, and neutropenia/lymphopenia. As shown in Fig 2, 86% and 67% of the patients treated respectively with Filgrastim or Exenatide were transplanted when small islet infusion volumes (< 5ml) were used. In order to try and dissociate the effect of the drug from the one of the infusion volume, the effect of Exenatide and/or Filgrastim treatment was estimated after adjusting for the dichotomized volume variable (infusion volume ≥ or < 5ml).

This analysis reveals that the association between graft survival and Exenatide remained statistical significant whereas significance was lost for Filgrastim or for Filgrastim and Exenatide once the infusion volume is considered (S1 Table and S2B Fig). Nevertheless the direction of the estimates for Filgrastim alone, Exenatide alone, and Exenatide plus Filgrastim treatment suggest protective effects against graft failure and warrant prospective randomized, controlled studies to further determine and eventually validate the role and mechanism of these agents in promoting long-term islet transplant function.

Another confounding factor is the administration of Filgrastim, alone or in combination with Exenatide, only in presence of neutropenia as standard of care treatment rather than investigational drug. This study did not include no-neutropenic Filgrastim treated patients and thus it is impossible to dissociate the role of neutropenia from the one of the drug in promoting allograft survival.

## Discussion

The retrospective analysis of our islet transplant experience indicates that the administration of Filgrastim and Exenatide in combination with moderate/severe neutropenia may have a positive effect on long–term islet transplant survival. Although, because of the retrospective nature of the analysis, there are many inherent confounding factors that complicate estimation of independent associations with graft survival: administration of both Filgrastrim and Exenatide as standard of care to ameliorate respectively moderate to severe neutropenia and post-prandial hyperglycemia; changes in the islet infusion volumes and induction treatment used and aimed to implement the transplant procedure. Besides, the association between the administration of Filgrastim and/or Exenatide and graft survival may be explained immunologically: Exenatide reduces pancreatic beta cell apoptosis [19], pancreatic T-cell infiltration [20], and inflammation [13,20]. The use of this GLP-1 analog, by favoring insulin release and reducing blood glucose level, may contribute to decrease extracellular ATP levels [21]. The reduced level of ATP may result in a decrease activation of the purinergic receptors P2X7R thus the increased release of pro-inflammatory cytokines and recruitment and activation of Th1/Th17 prodiabetogenic cells [22–24]. In vitro studies had shown that targeting of P2X7R with periodateoxidized ATP (oATP) during allo-stimulation inhibits the production of Th1/Th17 cytokines and T-cells activation [24]. Moreover the co-administration of oATP and P2X7R inhibitors delays islets allograft rejection [25].

Filgrastim recruits myeloid derived suppressor cells (MDSCs), expands regulatory T-cells (Treg), and improves allograft survival in immunocompetent mice [14]. Filgrastim may in fact positively impact allograft survival trough auto-immune disease prevention, and immunosuppressive and tolerogenic promotion via recruitment of MDSC and Treg. Our previous preclinical data show how in-vivo administration of G-CSF significantly delays allogeneic skin graft rejection [26]. Thus, it is possible that a brief administration of Filgrastim may promote allograft acceptance by inducing MDSC and Treg. Other immune players may be affected by Filgrastim administration. Transplanted islets associated dendritic cells (DC), that promote allograft rejection, may be differentiated towards a tolerogenic phenotype [27]. Moreover, previous pre-clinical data have shown that the depletion of islets donor associated DC promotes allograft survival [28].

Due to the retrospective nature of the analysis and to the fact that Filgrastim was administered as standard of care, we cannot rule out the possible effect of neutropenia itself on transplant acceptance. However, recent clinical trials in new onset T1D show that treatment with the neutropenic agent Anti-Thymocyte Globulin (ATG) together with PEGylated filgrastim [15] significantly delays the progression of diabetes. Interestingly, similar trials using only ATG did not show positive results suggesting that neutropenia and Filgrastim treatment might be necessary for the long-term tolerogenic effect. Beside its known role as incretin regulator [8], Exenatide was shown to inhibit cytokine induced beta cells apoptotic death [29]. Both Filgrastim and Exenatide may have important immunological actions that facilitate graft survival, and further investigation on the possible combinatorial effect of moderate/severe lymphopenia and Filgrastim and Exenatide administration as treatment to increase the success rate in islets transplantation is needed.

Another important aspect that needed to be considered was the possible effect of Sirolimus (rapamycin) as immunosuppressive maintenance agent. Although all of the 44 patients underwent initial immunosuppression maintenance with Sirolimus, because of the appearance of severe side effects such as ovarian cysts, mouth ulcers and peripheral edema in 6 patients, treatment with this mTOR inhibitor was suspended after a median of 5.56 years (range 0.81–7.76 years) and replaced with the calcineurin inhibitor Tacrolimus and mycophenolate mofetil (MMF). Although Kaplan Meyer analysis does not reveal any difference in graft rejection (logrank p = 0.495), it is not possible to exclude a beneficial initial impact of rapamycin treatment. Indeed, several pre-clinical studies have demonstrated the capacity of mTOR inhibitors to promote peripheral tolerance via Hematopoietic Stem Cells (HSCs) mobilization. For example, in preclinical models addition of rapamycin to anti-CXCR4 therapy was shown to promote PD-L1^+^HSC mobilization, induce a robust and transferable host hyporesponsiveness and promote islet allograft survival [30]. Furthermore, GM-CSF mobilized MDSCs like HSCs that stimulates peripheral Foxp3+Treg expansion [26,31,32], Similarly, GM-CSF induced Gr1^+^HSC phenotypically similar to MDSC were shown able to sustain peripheral immunological tolerance per se in autoimmune diabetes [33,34]. Considering that MDSCs are immature myeloid cells that derived and belong to the HSC we cannot exclude a possible synergistic effect on the mobilization and differentiation of these cells by Sirolimus and Filgrastrim. However, beside the possible role of Sirolimus alone or in combination with Filgrastin in promoting tolerogenic MDSCs it is important to remember that rapamycin was shown to promote Treg cells expansion and effector T cells depletion in PBMCs from T1D patients [35]. Thus multiple tolerogenic action may be played by this drug.

Our analysis also suggests the need to minimize intraportal infusion volume to reduce liver damage, while favoring islet survival. However, it is important to note that higher intraportal infusion volumes were used mostly in the initial years compared to more recent years (S2 Fig). Indeed, infusion volumes have decreased over the years as a result of the improvement of isolation and purification methods. This progressive reduction in infusion volumes has also paralleled an optimization of immunosuppressive protocols (e.g., improvement in islet processing, purification technology, and improved immunosuppression protocols) and thus it may explain this observation.

Induction strategies have been modified over the years to reduce adverse events and improve islet allograft survival. Particular attention had been given to the instant blood-mediated inflammatory reaction (IBMIR) and the consequent cytokine storm. This acute inflammatory reaction involves platelet and complement activation, and MCP-1 mediated infiltration of neutrophils, monocytes, and macrophages whose activation leads to the release of several pro-inflammatory cytokines including IL-1β, TNF-α, and IFN-γ that trigger STAT1 and NFKB mediated apoptosis in the transplanted islets during the early stages of transplantation [36,37] TNF-α represents one of the most dominant targets in acute inflammatory injury of islets. In accordance to the initial study by Farney et al. [38] showing benefits of TNF-α blockade in mice receiving syngeneic islet grafts and the subsequent clinical inclusion of Etanercept in a single-donor islet transplant protocol [39] NF-a blockade has been included as immunosuppressive agents in our and other’s transplant protocols [40,41]. However, the current retrospective analysis performed on our court does not confirm these data. Indeed, anti-TNF-α agents in association with anti-CD25 treatment not only did not appear to be beneficial, but also may have negatively affected graft survival [4]. Interestingly, high levels of TNF-α were shown to inversely correlate with immune activation [42] in healthy donors and in patients with type 2 diabetes. Furthermore, during chronic inflammation, TNF-α contributes to arrest MDSC differentiation and enhances their immune regulatory activity [43]. TNF-α blockade during early chronic inflammatory will thus results in MDSCs’ inhibition and maturation into dendritic cells and macrophages, favoring immunoactivation [43] and graft rejection. In view of the growing literature suggesting a tolerogenic role of TNF-α, Etanercept use in induction protocol should be further evaluated.

Although the power of our analysis was not sufficient, Alemtuzumab induction might be superior to Daclizumab, suggesting the need for further investigation on the use of Alemtuzumab. Indeed, Alemtuzumab was previously associated with a profound immunosuppression, depletion of T-cells and B-cells, but with a transient increase in Treg that may promote graft survival [14]. On the contrary, Daclizumab can deplete not only effector cells but also Treg [15] by binding to the high affinity IL-2 receptor CD25 [44].

In conclusion, our experience in islet transplantation confirms the clinical benefits of this procedure and highlights the need for prospective placebo-controlled randomized clinical trials to further optimize induction protocols and to test the efficacy of short immune modulatory intervention using Exenatide and Filgrastim.

## Supporting Information

S1 Fig Effect of different induction regimens on allograft survival. **A)** Comparison of the effect of induction with Daclizumab alone or in combination with Etanercept anti-TNF-α treatments. **C)** Comparison of allograft survival obtained using Daclizumab or Alentuzumab in combination with anti-TNF-α treatments or with Infliximab. **B)** Allograft survival obtained with Daclizumab alone or in combination with all.
(DOCX)

S2 Fig Changes of infusion volume over the years. **A)** In the attempt to reduce liver damages while promoting islets survival, volume of the first infusion were reduced from 5ml and above to less than 5ml. **B)** Effect of Exenatide and/or Filgrastim treatment adjusted for the dichotomized volume variable (infusion volume < 5ml).
(DOCX)

S1 Table Cox regression model used to determine the effect the indicated variable on allograft survival. (DOCX)
