# Supplementary material for: G-CSF and Exenatide Might Be Associated with Increased Long-Term Survival of Allogeneic Pancreatic Islet Grafts
Source: PLoS One. 2016 Jun 10;11(6):e0157245. doi: 10.1371/journal.pone.0157245 (PMC4902232; doi:10.1371/journal.pone.0157245)
Supplement: S1 Fig — A) Comparison of the effect of induction with Daclizumab alone or in combination with Etanercept anti-TNF-α treatments. C) Comparison of allograft survival obtained using Daclizumab or Alentuzumab in combination with anti-TNF-α treatments or with Infliximab. B) Allograft survival obtained with Daclizumab alone or in combination with all. (DOCX) [file pone.0157245.s001.docx]

**S1_Figure**


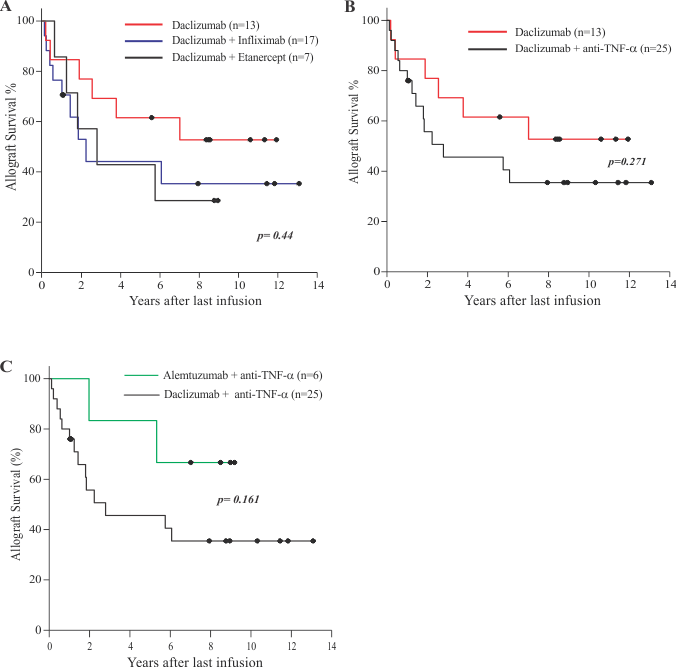


**S1 Figure: Effect of different induction regimens on allograft survival. A)** Comparison of the effect of induction with Daclizumab alone or in combination with Etanercept anti-TNF-α treatments. **C)** Comparison of allograft survival obtained using Daclizumab or Alentuzumab in combination with anti-TNF-α treatments or with Infliximab. **B)** Allograft survival obtained with Daclizumab alone or in combination with all.
