# Supplementary material for: G-CSF and Exenatide Might Be Associated with Increased Long-Term Survival of Allogeneic Pancreatic Islet Grafts
Source: PLoS One. 2016 Jun 10;11(6):e0157245. doi: 10.1371/journal.pone.0157245 (PMC4902232; doi:10.1371/journal.pone.0157245)
Supplement: S2 Fig — A) In the attempt to reduce liver damages while promoting islets survival, volume of the first infusion were reduced from 5ml and above to less than 5ml. B) Effect of Exenatide and/or Filgrastim treatment adjusted for the dichotomized volume variable (infusion volume < 5ml). (DOCX) [file pone.0157245.s002.docx]

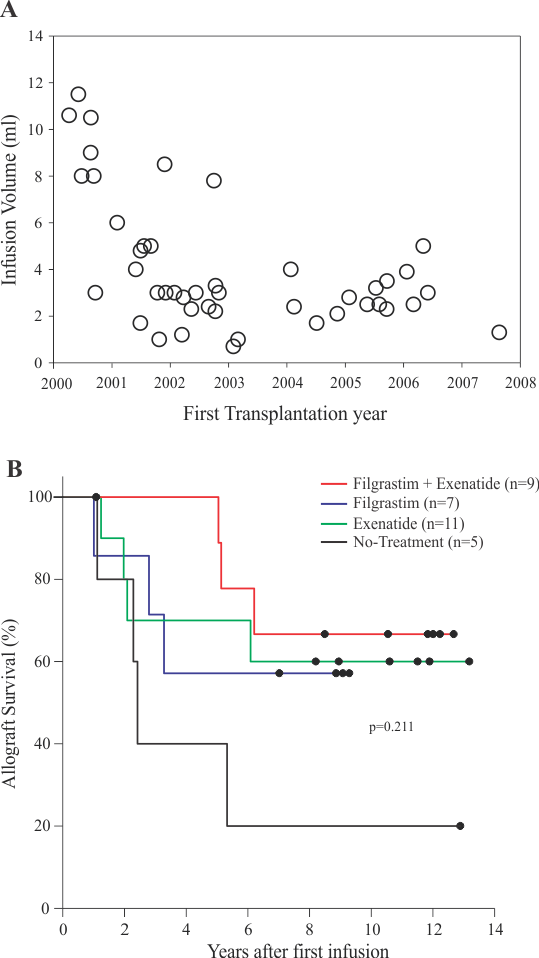
**S2 Figure**

**S2_Figure: Changes of infusion volume over the years. A)** In the attempt to reduce liver damages while promoting islets survival, volume of the first infusion were reduced from 5ml and above to less than 5ml. **B)** Effect of Exenatide and/or Filgrastim treatment adjusted for the dichotomized volume variable (infusion volume < 5ml).
