# Supplementary material for: G-CSF and Exenatide Might Be Associated with Increased Long-Term Survival of Allogeneic Pancreatic Islet Grafts
Source: PLoS One. 2016 Jun 10;11(6):e0157245. doi: 10.1371/journal.pone.0157245 (PMC4902232; doi:10.1371/journal.pone.0157245)
Supplement: S1 Table — (DOCX) [file pone.0157245.s003.docx]

**S1 Table**

| **Sup.Table.S1: Cox regression model used to determine the effect the indicated variable on allograft survival.** | | | | | |
| --- | --- | --- | --- | --- | --- |
| **Variable** | **Regression Coefficient** | **Exp^(Coefficient)^** | **SEM** | **Z** | ***p*** |
| Cox regression model obtained considering the different drug treatment administered on allograft survival. | | | | | |
| Exenatide only | -1.07 | 0.344 | 0.518 | -2.06 | 0.039 |
| Filgrastim only | -1.24 | 0.290 | 0.678 | -1.83 | 0.068 |
| Exenatide and Filgrastim | -1.46 | 0.232 | 0.683 | -2.14 | 0.032 |
| Likelihood ratio test=6.8 on 4 df, *p=0.0785* n=44, number of event=22 | | | | | |
|  | | | | | |
| Cox regression model obtained considering dichotomized variables: Volume of first infusion and drug treatment administered. | | | | | |
| Volume of the First Infusion | 0.681 | 1.976 | 0.520 | 1.31 | 0.190 |
| Exenatide only | -0.956 | 0.384 | 0.531 | -1.80 | 0.0072 |
| Filgrastim only | -0.919 | 0.399 | 0.739 | -1.24 | 0.210 |
| Exenatide and Filgrastim | -1.148 | 0.317 | 0.743 | -1.55 | 0.120 |
| Likelihood ratio test=8.49 on 4 df, *p=0.0072* n=44, number of event=22 | | | | | |
